# Preperitoneal pelvic balloon tamponade—an effective intervention to control pelvic injury hemorrhage in a swine model

**DOI:** 10.3389/fbioe.2024.1340765

**Published:** 2024-04-26

**Authors:** Xiaogao Jin, Qinjun Chu, Hailong Bing, Fang Li, Jingyue Bai, Junge Lou, Liwei Sun, Chenxi Zhang, Lin Lin, Liumei Li, Haibo Wang, Zhanfeng Zhou, Junfeng Zhang, Hongkai Lian

**Affiliations:** ^1^ Department of Anesthesiology, The Second Affiliated Hospital of Guangdong Medical University, Zhanjiang, Guangdong, China; ^2^ Department of Anesthesiology and Perioperative Medicine, Zhengzhou Central Hospital Affiliated to Zhengzhou University, Zhengzhou, China; ^3^ Department of Orthopedics, Zhengzhou Central Hospital Affiliated to Zhengzhou University, Zhengzhou, China; ^4^ Department of Peripheral Vascular Intervention, Zhengzhou Central Hospital Affiliated to Zhengzhou University, Zhengzhou, China; ^5^ Department of Ultrasound Medicine, Zhengzhou Central Hospital Affiliated to Zhengzhou University, Zhengzhou, China; ^6^ Department of Anesthesiology, Tongren Hospital, Shanghai Jiao Tong University School of Medicine, Shanghai, China; ^7^ Research of Trauma Center, Zhengzhou Central Hospital Affiliated to Zhengzhou University, Zhengzhou, China

**Keywords:** pelvic fracture, hemorrhage, unstable hemodynamics, preperitoneal packing, balloon

## Abstract

**Objective:** This study aimed to estimate the effects of the volume of preperitoneal balloon (PPB) on arterial and venous hemorrhage in a swine pelvic fracture model.

**Methods:** Twenty-four swine were randomized into 0-mL, 500-mL, 800-mL, and 1000-mL intra-hematoma PPB groups. They were subjected to open-book pelvic fracture and reproducible injuries in the external iliac artery and vein. The pelvic binder and IH-PPBs with different volumes of fluid were applied to control the active hemorrhage after arterial and venous injuries. The survival time and rate during 60-min observation and digital subtraction angiography (DSA) images were the primary endpoints in this study. Secondary endpoints included survival rate within 70 min, peritoneal pressure, hemodynamics, blood loss, infusion fluid, blood pH, and lactate concentration.

**Results:** Our results indicated that the 800-mL and 1000-mL groups had a higher survival rate (0%, 50%, 100% and 100% for 0, 500, 800, and 1000-mL groups respectively; *p* < 0.0001) and longer survival time (13.83 ± 2.64, 24.50 ± 6.29, 55.00 ± 6.33, and 60.00 ± 0.00 min for 0, 500, 800, and 1,000 groups respectively; *p* < 0.0005) than the 0-mL or 500-mL groups during the 60 min observation. Contrastingly, survival rate and time were comparable between 800-mL and 1000-mL groups during the 60-min observation. The IH-PPB volume was associated with an increase in the pressure of the balloon and the preperitoneal pressure but had no effect on the bladder pressure. Lastly, the 1000-mL group had a higher mean arterial pressure and systemic vascular resistance than the 800-mL group.

**Conclusion:** IH-PPB volume-dependently controls vascular bleeding after pelvic fracture in the swine model. IH-PPB with a volume of 800 mL and 1000 mL efficiently managed pelvic fracture-associated arterial and venous hemorrhage and enhanced survival time and rate in the swine model without evidences of visceral injury.

## Introduction

Hemodynamically unstable pelvic fracture is characterized by a markedly high mortality rate, attaining 40% due to uncontrollable bleeding (Parry et al., 2021; Yakkanti et al., 2021; McDonogh et al., 2022). 80% of hemorrhages related to pelvic fracture are hypothesized to originate from the spongy bone and the venous plexus, with only 20% of hemorrhages resulting from the injured artery (Lee et al., 2020; Mejia et al., 2020). At present, both preperitoneal packing and angioembolization have been established as efficient strategies for the management of fatal hemorrhages resulting from severe pelvic fracture (Burlew et al., 2017; Sandhu et al., 2020; McDonogh et al., 2022). However, the time from patient arrival to angioembolization was documented to be as long as 75 min, even in the hybrid emergency room system (Ito et al., 2020). Reports on the application of preperitoneal packing or angioembolization following serious pelvic fractures are controversial (Cheng et al., 2018; Kam et al., 2019). The former is associated with swift hemorrhage control in pre-hospital or in-hospital settings (Li et al., 2016). The traditional open preperitoneal packing typically requires a sterile environment and is unsuitable for use outside the operation room. Recently, Dr. Do et al. have successfully employed a minimally invasive preperitoneal balloon to manage the pelvic fracture-associated hemorrhage in a swine model (Sokol et al., 2016; Do et al., 2019a; Do et al., 2019b). Compared to conventional preperitoneal packing, PPB offers benefits in terms of ease of placement and the ability to be deflated during subsequent embolization (Sokol et al., 2016; Do et al., 2019a; Do et al., 2019b; Mikdad et al., 2020; Asmar et al., 2021). Moreover, it is minimally traumatic following successful resuscitation compared with surgical packs generally used in open preperitoneal packing.

Consequently, PPB is a potential method that may substitute traditional open preperitoneal packing in the future. Herein, PPB was applied to manage bleeding after vascular injury in a severe pelvic fracture swine model that was developed by Dr. Do. However, Dr. Do did not show how much volume of PPB is required to control the hemorrhage in the model of pelvic fracture. Moreover, a large hematoma has already formed after injury of the external iliac vessels while attempting to insert the PPB into the preperitoneal space. The hematoma was anticipated to be removed in the traditional preperitoneal packing (Colacchio et al., 2009; Filiberto and Fox, 2016; Lin et al., 2021). Nevertheless, this process posed challenges in maintaining stable blood pressure during hematoma removal. Interestingly, draining the hematoma using preperitoneal packing did not achieve hemorrhage control and conversely disrupted hemodynamical stability. Therefore, we hypothesized that an intra-hematoma preperitoneal balloon may efficiently control bleeding by compressing vessels along with the hematoma. Thus, this study aimed to identify the optimal volume of IH-PPB along with the existed hematoma to effective control of arterial and venous hemorrhage in a modified swine model.

## Methods

### Animal and anesthesia

Twenty-four white swine, weighing 35–45 kg and aged 8-9 months were randomized to four groups, including the 0-mL, 500-mL, 800-mL, and 1000-mL IH-PPB groups. The adult pelvic volume within bones is approximately 1 L in human beings (Baque et al., 2005) and below 100–200 mL in swine (weight 35–45 kg). However, the external iliac artery and vein were injured following pelvic fracture in the swine model in this study, and blood accumulated in the preperitoneal space rather than the pelvic cavity. In the swine model employed herein, the unilateral potential preperitoneal space was estimated to have a capacity of roughly 1 L. Overall, a pelvic facture with external iliac vessels injuries was used to simulate serious pelvic fractures of human being in this study. Intramuscular atropine sulfate (0.04 mg/kg) and buprenorphine (0.02 mg/kg) were administered to induce anesthesia. Following this, an endotracheal tube was inserted after intramuscular administration of esketamine (10 mg/kg) and midazolam (400 μg/kg). Anesthesia was maintained through the inhalation of sevoflurane (1%–3%) and intravenous infusion of propofol (1–4 mg/kg/h). Whilst neuromuscular blockade was achieved by continuous infusion of cis-atracurium (0.2–0.6 mg/kg/h) (Tutunaru et al., 2017).

### Hemodynamical monitoring setup and preparation of vascular injuries

The pelvic fracture model with vascular injuries was constructed according to the methodology outlined by Dr. Do (Do et al., 2019a). Briefly, the right carotid artery and internal jugular vein were cannulated with an 8F catheter (5F*11 cm, Cordis Corporations, United States) under ultrasonic guidance to measure arterial and venous pressure, respectively. The left internal jugular vein was cannulated using an 8.5 Fr kit for rapid infusion and a Swann-Ganz catheter (7.5 F × 110 cm, Edwards Lifesciences LLC, United States) was placed through the 8.5 F catheter (8.5 F × 10 cm, Edwards Lifesciences LLC, United States). An 8 Fr Cordis catheter was also placed into the left internal jugular vein to facilitate the placement of a vascular balloon for venous angiography.

**FIGURE 1:**
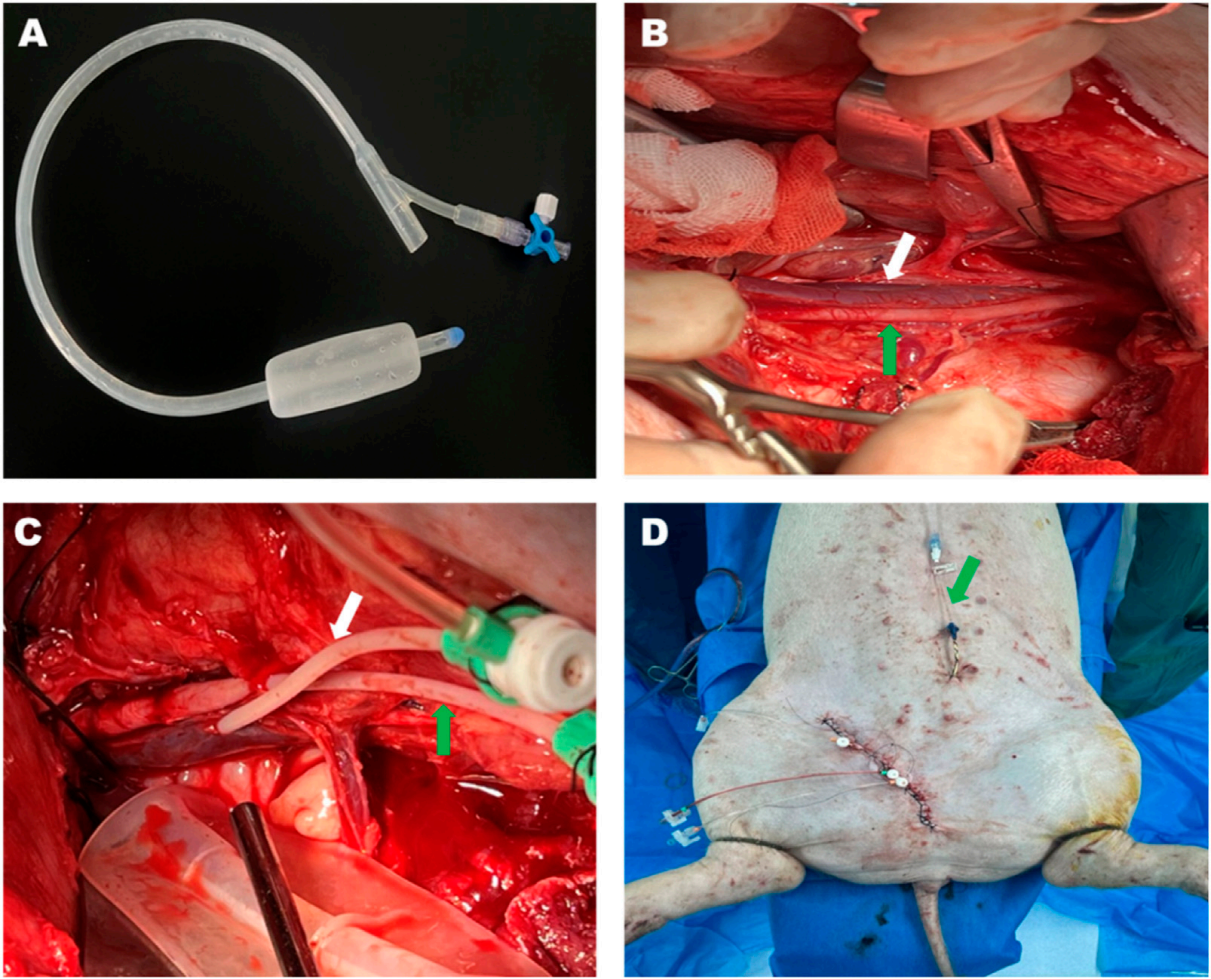
Bakri postpartum balloon and the process of the animal experiment. **(A)** The Bakri postpartum balloon was used in this study. The Bakri balloon was placed beside the injured vessels in the potential preperitoneal space under the field of vision. **(B)** The anatomy of the left external iliac artery (green arrow) and vein (white arrow) after operational exposure. **(C)** The external iliac artery (green arrow) and vein (white arrow) were cannulated using 5-F catheters. **(D)** The surgical intervention was terminated before vessel injury. The green arrow pointing toward the suprapubic cystostomy.

A 10-cm incision was made above the right inguinal crease to expose the external iliac artery and vein. Next, a 5-Fr Catheter was, respectively, inserted into the external iliac artery and vein using the Seldinger technique. Following this, 5-mm vascular dilators (5 mm, NC Sprinter™, Rapid Exchange Balloon Dilatation Catheter, Medtronic Inc., United States) were placed in the external iliac artery and vein through the 5-F catheters (5 F × 11 cm, Cordis corporation, United States) to reproduce similar injuries in the anterior walls of the artery and vein (Figure 1).

### Establishment of pelvic fracture model

The Bakri postpartum balloon (24F, Henan Jianhe Industry, LLC, China) was positioned at a minimum 5 cm medially from the vessels that were supposed to be injured (Figure 1). The drain termination of the balloon was connected to a pressure transducer (Biotrans II Kit 859, Biobtimal International PTE. Ltd., Singapore) to monitor the preperitoneal pressure. The left femoral vein was cannulated with a 5F catheter (5 F × 11 cm, Cordis Corporation, United States) to inject contrast media during angiography when the inferior vena cava was occluded by an endovascular Occlusion Balloon (Embolectomy Catherter Fogarty^R^, 7Fr, 80 cm, Edwards Lifesciences, US). Lastly, the wound was meticulously closed by running sutures to prevent blood leakage while leaving the tail of vascular dilators externally to elicit vascular injury.

A vertical 4-cm incision was made over the pubic symphysis to expose the pubic symphysis, which was subsequently separated from the midline using a bone chisel. Then, a Finochietto rib spreader was introduced into the separated site to extend the diastasis to 3 cm. The sacroiliac joints and posterior ligaments were mobilized along the separated span of the pubic symphysis. Afterward, a 3-cm wooden block was inserted in the separated site to maintain the diastasis throughout the whole experiment. The incision was closed using multilayer running sutures. Suprapubic cystostomy was performed under ultrasonic guidance to collect urine and measure the bladder pressure.

A pigtail catheter was inserted into the left carotid artery through an 8Fr catheter, and its tip was placed proximal to the aortic bifurcation, covering both the right and left iliac arteries for arterial angiography. At the same time, the endovascular occlusion balloon was inserted into the left internal jugular vein and positioned at the tip, proximal to the bifurcation of the inferior vena cava for venous angiography.

### Experimental procedure

The experiment was initially upon the removal of vascular dilators along with 5-Fr catheters. Angiography was performed to confirm the vascular injury after pulling out of the vascular dilator. The pelvis was immediately wrapped by a long drape to control pelvic volume. The pelvis was thereupon wrapped by the drape to let the bladder pressure lower than 5 mmHg to avoid visceral injury. The swine’s legs were securely tied crossly after the mean arterial pressure dropped below 40 mmHg. The IH-PPB, which was prepositioned in the preperitoneal space, was immediately inflated to maintain the MAP above 40 mmHg. Hextend was infused to maintain a mean arterial pressure over 40 mmHg when the MAP dropped below 40 mmHg. Although colloid as resuscitative fluids do not significantly improve the mortality rate, they are speculated to mitigate hemodynamically instability (Heming et al., 2017; Gordon and Spiegel, 2020). Thus, 6% Hetastarch in Lactated Electrolyte Injection was administered following severe shock. Resuscitation efforts were halted if MAP exceeded 40 mmHg. It is worthwhile noting that the normal MAP ranges from 80 to 120 mmHg from the swine used in this study (Hwang et al., 2016).

Angiography was performed using iodixanol (Yangtze River Pharmaceutical Co., Ltd, China) at a rate of 20 mL/s under a pressure of 600 psi through the line previously placed in the left femoral artery or carotid artery or internal jugular vein. Of note, angiography was performed immediately after vascular injury and within 5 min of balloon inflation. The contrast media extravasation was considered a positive result in the angiograph.

Following a 60-min observation period, the balloon was deflated, and the survival animal was monitored for another 10 min. Heart rate and MAP were additionally recorded every 1 min during the experiment. The swine was euthanized at a MAP of 20 mmHg or 70 min after vascular injury, whichever occurred first. After euthanasia, blood and clots were extracted from the preperitoneal space and measured as total blood loss (TBL) by weight (1 g≅1 mL). The bleeding rate and infusion rate was calculated by the total blood loss or total infusion being divided by the survival time. Laparotomy was also performed on all animals after euthanasia to assess the possible damage to the peritoneum, colon, bladder, and rectum. The euthanasia was carried out by rapid intravenous injection of 10% Potassium chloride (KCl) 10 mL, followed by Incision of the chest cavity to produce a pneumothorax and cessation of respiration.

### Statistical analysis

Continuous data following normal distribution were presented as mean and standard deviation (Mean ± SD) in this study. Survival rate and positive rate in angiography were compared using the X^2^ test or fisher’s exact testing. Continuous variables between the groups were compared by one-way analysis of variance (ANOVA). A two-way ANOVA was employed to analyze data for MAP (Mean Arterial Pressure), CI (Cardiac Index), PCWP (Pulmonary Capillary Wedge Pressure), HR (Heart Rate), and SVR (Systemic Vascular Resistance) while adjusting for the effects of repeated measures. *p* < 0.05 was considered as statistically significance.

## Results

All twenty-four white swine were included in the analysis, and no accident or death was reported. The baseline weight, hemodynamic, bladder pressure, preperitoneal space pressure, lactate concentration, and plasma pH value were comparable among the four groups (Table 1).

**TABLE 1:** Baseline data before the experiment in the groups (Mean ± SD).

Variables	0-mL	500-mL	800-mL	1000-mL	*p*-value
N	6	6	6	6	NA
Weight (kg)	38.67 ± 4.92	39.17 ± 2.32	39.5 ± 2.74	38.5 ± 4.0	0.9621
Map (mmHg)	86.3 ± 6.0	82.5 ± 6.1	87.7 ± 6.4	83.8 ± 4.1	0.4120
HR (beat/min)	82.17 ± 6.34	83.17 ± 7.76	84.33 ± 5.72	83.67 ± 5.72	0.9417
PCWP (mmHg)	8.50 ± 1.87	9.17 ± 1.33	8.83 ± 1.72	8.33 ± 1.97	0.8456
HCT (%)	23.33 ± 2.25	21.50 ± 2.26	22.33 ± 2.07	23.00 ± 1.41	0.4323
Preperitoneal pressure (mmHg)	0	0	0	0	NA
Bladder pressure (mmHg)	5.67 ± 1.97	4.83 ± 2.14	6.00 ± 2.19	5.33 ± 2.25	0.8074
CVP (mmHg)	2.50 ± 1.05	1.83 ± 1.47	2.17 ± 1.47	2.00 ± 1.41	0.8522

Note: NA, not applicable.

**FIGURE 2:**
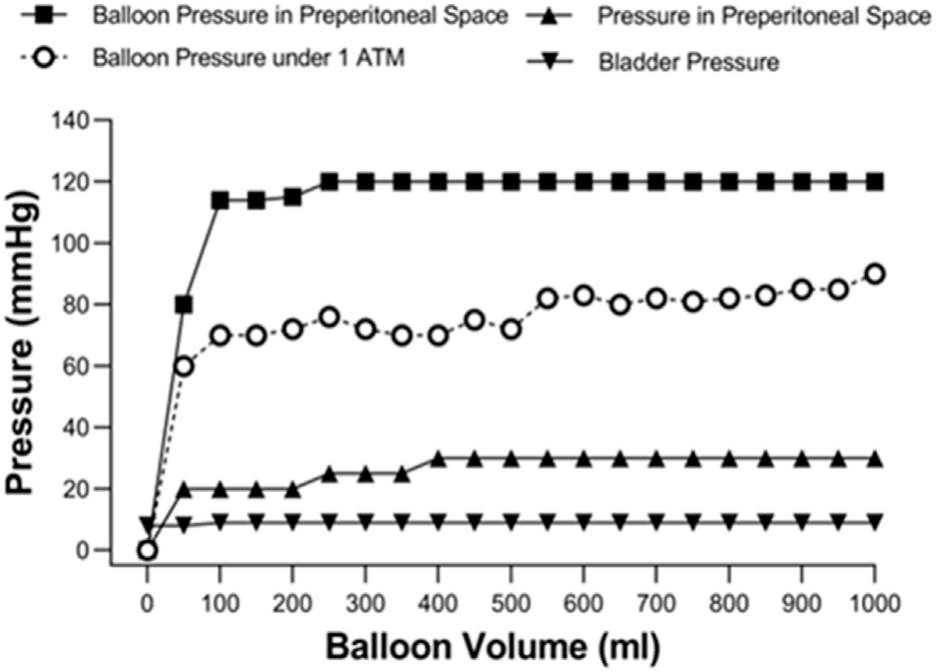
Figure-relationship between balloon volume and the pressure-related parameters under atmospheric pressure (1ATM) and the preperitoneal space without vascular injury and pelvis wrapping.

Analyzing the pressure of the Bakri balloon and the fluid volume revealed that the balloon pressure remained stable at 80–90 mmHg when inflated with 200–1,000 mL of normal saline under atmospheric pressure (1 ATM) (Figure 2). To understand the relationship between balloon volume and preperitoneal pressure, the preperitoneal pressure was measure after preperitoneal balloon was inflated from 0 mL to 1000 mL in normal animal without vascular injury and pelvis wrapping. The pressure of the Bakri balloon was increased and maintained at 110–125 mmHg in the preperitoneal space of the swine without vascular injury. The preperitoneal pressure was maintained at 25–30 mmHg when the balloon was inflated with 200–1,000 mL of normal saline. Our preliminary experiments also determined that Bladder pressure (8-9 mmHg) was not correlated with the volume of the Bakri balloon.

**FIGURE 3:**
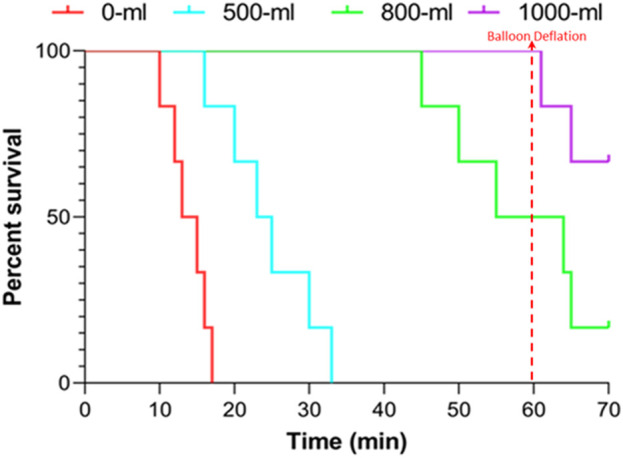
Survival rate changes over time in the four groups after vascular injury.

The survival time were 13.83 ± 2.64, 24.50 ± 6.29, 55.00 ± 6.33, and 60.00 ± 0.00 min for the 0-mL, 500-mL, 800-mL, and 1000-mL groups within the 60-min observation period following vascular injury, respectively (500-mL vs. 0-mL group, *p* = 0.0038; 800-mL vs. 0-mL group, *p* < 0.0001; 1000-mL vs. 0-mL group, *p* < 0.0001; 800-mL vs. 500 mL group, *p* < 0.0001; 1000-mL vs. 500-mL group, *p* < 0.0001; 000-mL vs. 800-mL group, *p* = 0.2751). Meanwhile, the survival rates were 0%, 50%, 100%, and 100% for the 0-mL, 500-mL, 800-mL, and 1000-mL groups over 60 min observation, respectively (*p* < 0.0005). Moreover, the survival results signaled that the 800-mL and 1000-mL groups had a higher survival rate and longer survival time compared with those of the 0-mL and 500-mL groups during the 60-min observation period (Figure 3). In the additional 10-min observation after balloon deflation, survival rates were similar between the 800-mL and 1000-mL groups (Table 2), suggesting that 800-mL and 1000-mL IH-PPB had equivalent efficacy in improving the survival rate of swine in this study.

**TABLE 2:** Survival situation after an additional 10-min observation (Fisher’s exact test).

Groups	Alive	Dead	*P*
800-mL	1	5	0.12
1000-mL	4	2	

**FIGURE 4:**
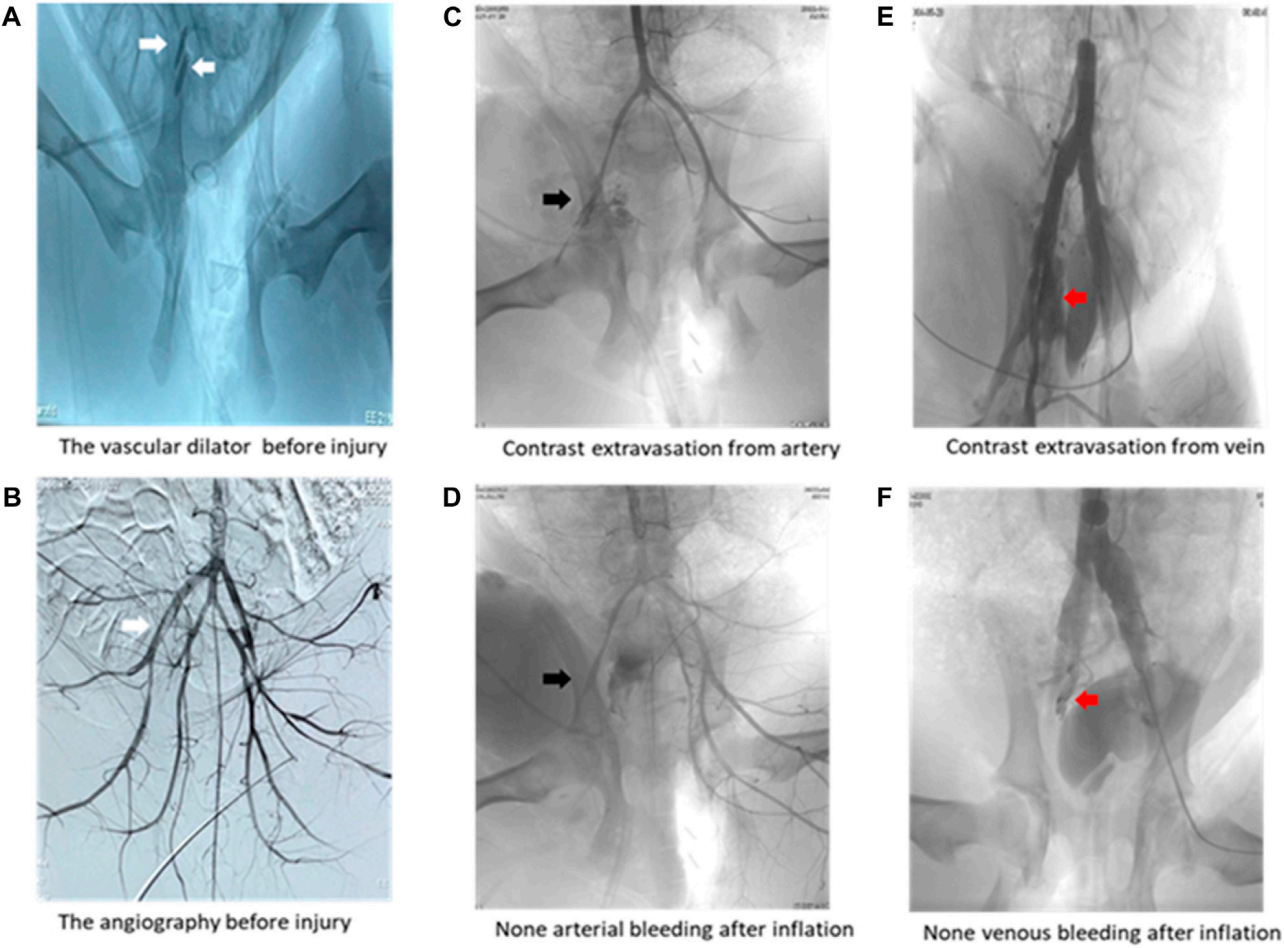
Representative angiography images. **(A)** Two white arrows pointing toward the vascular dilator in the left external iliac artery and vein. **(B)** White arrow pointing toward the vascular dilator in the left external iliac artery. **(C)** Black arrow pointing toward contrast media extravasation from the external iliac artery. **(D)** Black arrow displaying that IH-PPB prevented bleeding from the external iliac artery. **(E)** Red arrow illustrating contrast media extravasation from the external iliac vein. **(F)** Red arrow depicting that IH-PPB prevented bleeding from the external iliac vein.

Arterial angiography results (Table 3) demonstrated that the1000-ml group had a lower positive rate in arterial and venous angiography than the 0-mL and 500-mL groups (Table 3). Additionally, the 800-mL group had a lower positive rate in venous angiography than the 0-mL or 500-mL groups. The angiography results confirmed that 800-mL and 1000-mL IH-PPB had equivalent efficacy in the management of vascular bleeding in swine (Figure 4).

**TABLE 3:** Angiograph of arteries and veins in pelvic cavity.

Groups	Positive result in arteries	Positive result in vein
0-mL (*n* = 6)	6	6
500-mL (*n* = 6)	5	3
800-mL (*n* = 6)	3	1^&^
1000-mL (*n* = 6)	0^@,#^	0^$^

^@^ *p* = 0.0022, compared to the 0-mL group; ^#^ *p* = 0.0152, compared to the 500-mL group; ^&^ *p* = 0.0152, compared to the 0-mL group; ^$^ *p* = 0.0022, compared to the 0-mL group.

**FIGURE 5:**
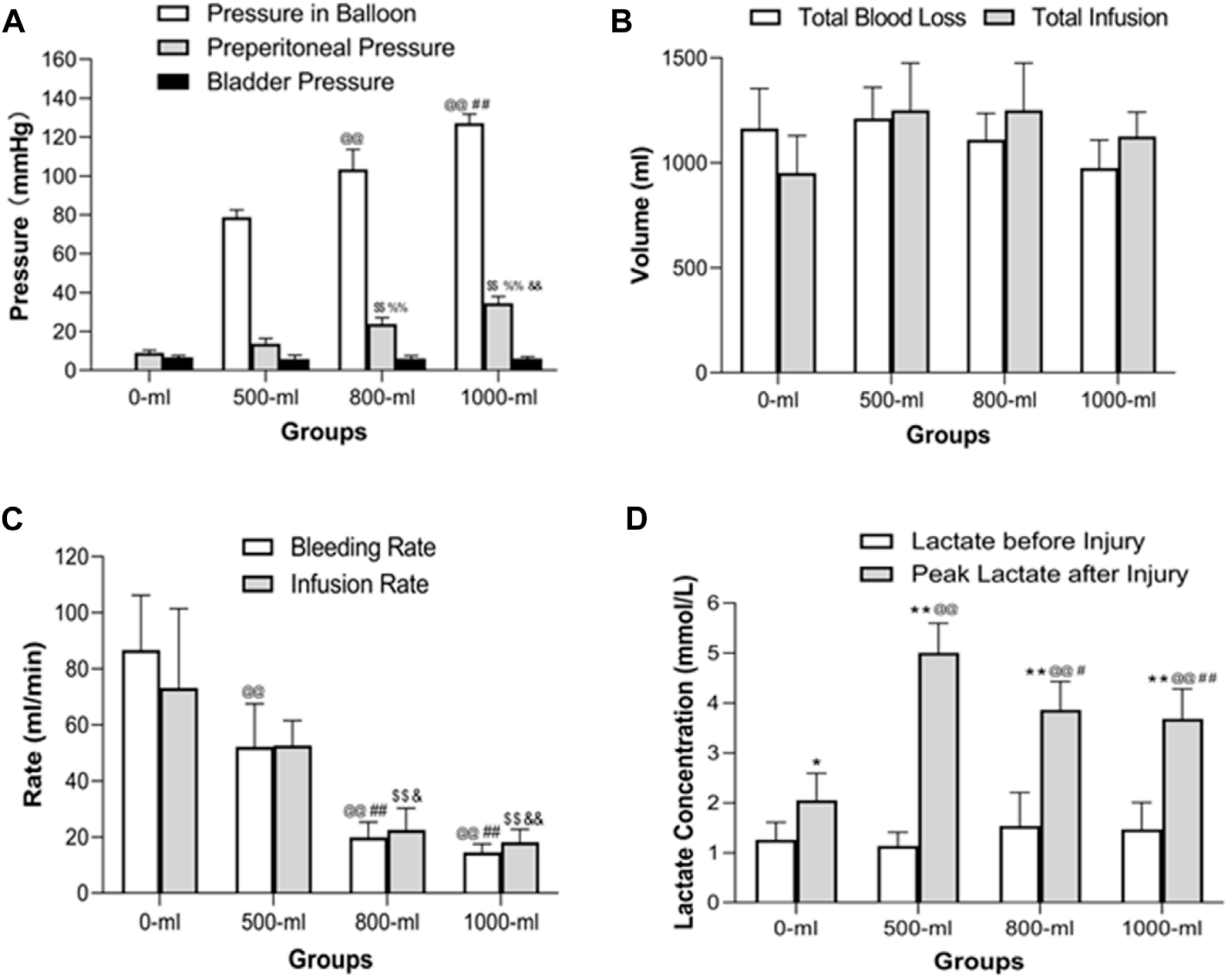
Intraballoon pressure, preperitoneal pressure, bladder pressure, total blood loss volume, total infusion volume, bleeding rate, infusion rate, and plasma lactate concentration in the four groups **(A)** Intraballoon pressure, preperitoneal pressure, and bladder pressure in the four groups. @@*p* < 0.001, compared to the 500-mL group; ##*p* < 0.001, compared to the 800-mL group; $$*p* < 0.001, compared to the 0-mL group; %%*p* < 0.001. **(B)** Total blood loss and total infusion volumes in the four groups. TBL was measured in the preperitoneal space after euthanasia. There were no significant differences in total blood loss and total infusion volumes. **(C)** The bleeding and infusion rates in the four groups. @@*p* < 0.001, compared to the 0-mL group; ##*p* < 0.001, compared to the 500-mL group; $$*p* < 0.001, compared to the 0-mL group; &*p* < 0.05, compared to the 500-mL group; &&*p* < 0.001, compared to the 500-mL group. **(D)** Plasma lactate levels in the four groups. *p < 0.05 (**p < 0.01) compared to Lactate levels before injury; @@*p* < 0.001, compared to the 0-mL group; ##*p* < 0.001 (#*p* < 0.05)), compared to the 500-mL group.

As anticipated, the pressure of the balloon in the 1000-mL group was higher than that in the 800-mL, 500-m, and 0-mL groups. Importantly, the 800-mL group had a higher balloon pressure than the 500-mL and 0-mL groups (Figure 5A). Similarly, the preperitoneal pressure in the 1000-mL group was higher than that in the 800-mL, 500-m, and 0-mL groups. The 800-mL group had a higher preperitoneal pressure than the 500-mL and 0-mL groups. Taken together, these results implied that IH-PPB volume played a critical role in survival rate by increasing the pressure in the preperitoneal space.

Remarkably, IH-PPB volumes ranging from 0 to 1,000 mL had a marginal effect on bladder pressure, indicating that IH-PPB volumes within this range do not result in significant organ damage within the pelvic space. Given the anatomical positioning of the bladder in proximity to the preperitoneal space, it may be displaced toward the opposite side when a preperitoneal balloon is placed on the contralateral side. Significant transmission of preperitoneal pressure occurs only if both sides of the preperitoneal space are occupied.

There were no significant differences in total blood loss and total infusion volumes among the four groups (Figure 5B). However, the 1000-mL and 800-mL groups had a lower bleeding rate and infusion rate than the 0-mL and 500-mL groups (Figure 5C). Meanwhile, the 500-mL group had a lower bleeding rate than the 0-mL group. These results collectively indicated that 800-mL and 1000-mL IH-PPB had comparable efficacy in controlling bleeding in the swine model. Nevertheless, attributed to the limited sample size in the current study, there may be insufficient statistical power to detect differences in hemorrhage and IVF volumes among the 4 subgroups.

Of note, the peak lactate concentrations after vascular injury were higher than the lactate concentration before injury in all four groups (Figure 5D). The 1000-mL and 800-mL groups had a lower lactate concentration after vascular injury compared with the 500-mL group. In addition, the 500-mL group had lower pH values than the 0-mL or 1000-mL groups. Remarkably, the pH was decreased only in the 500-mL group after vascular injury. We postulate that the swine in the 0-mL group experienced mortality before an elevation in lactate levels following vascular injury.

**FIGURE 6:**
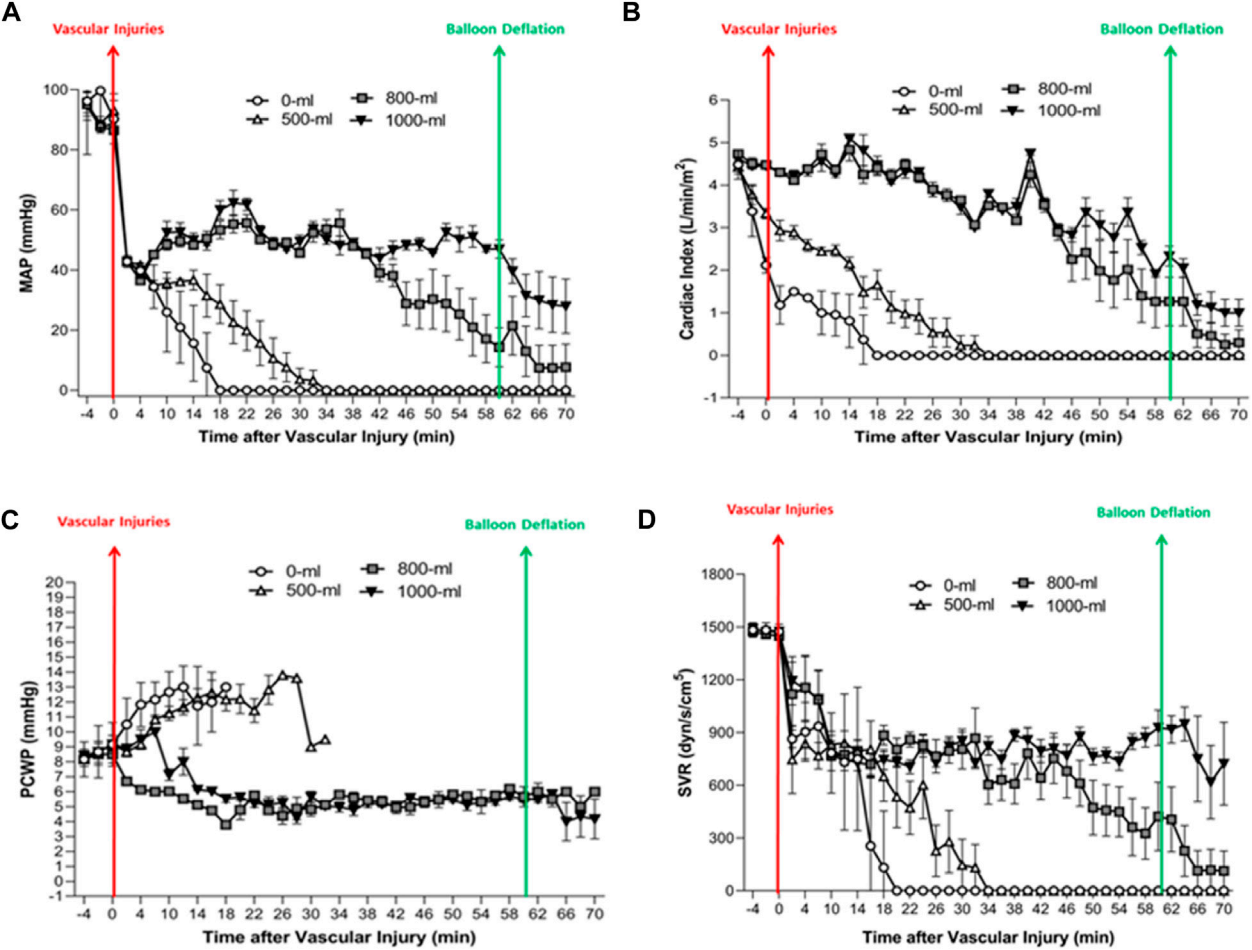
The MAP, CI, PCWP, and SVR change over time in the four groups. **(A)** Fluctuations in MAP (Mean Arterial Pressure) over time in the four groups. **(B)** Alterations in CI (Cardiac Index) over time in the four groups. **(C)** Changes in PCWP (Pulmonary Capillary Wedge Pressure) over time in the four groups. **(D)** Changes in SVR (Systemic Vascular Resistance) (mmHg/L/min; dyn/s/cm-5) over time in the four groups.

The 1000-mL and 800-mL groups had a higher MAP after vascular injury than the 0-mL and 500-mL groups (Figure 6A). Specifically, swine in the 1000-mL group had a higher MAP between 44 and 70 min after vascular injury compared to the 800-mL group. These observations implied that the increase in MAP after vascular injury was correlated with the volume of IH-PPB. Indeed, the use of 1,000 mL of IH-PPB was superior to 800 mL of IH-PPB in enhancing MAP after vascular injury.

The 1000-mL and 800-mL groups had higher CI values after the vascular injury compared to the 0-mL and 500-mL groups (Figure 6B), signifying that 1000-mL IH-PPB had exerted an effect similar to 500-mL IH-PPB in preserving cardiac functions after vascular injury.

The 0-mL and 500-mL groups had a higher PCWP after vascular injury compared with the 1000-mL and 800-mL groups (Figure 6C). It is worthwhile pointing out that PCWP significantly increased before cardiac arrest in the swine model if resuscitation efforts failed. Besides, 1000-mL IH-PPB had a similar capacity as 800-mL IH-PPB in maintaining PCWP after vascular injury.

The 1000-mL and 800-mL groups had a higher SVR between 22 min and 70 min after vascular injury compared with the 0-mL and 500-mL groups (Figure 6D). Likewise, the 1000-mL group had a higher SVR within the range of 46 and 70 min after vascular injury compared with the 800-mL group. These results demonstrated that the increase in SVR after vascular injury was correlated with IH-PPB volume and that 1000-mL IH-PPB outperformed 800-mL IH-PPB in improving SVR after vascular injury.

Necropsy showed no evidence of bowel and bladder injuries in all animals. Bowel and bladder biopsies also exhibited no signs of positive discovery under the microscope after HE staining (data not shown).

## Discussion

To the best of our knowledge, this is the first study to outline that IH-PPB controlled vascular hemorrhage in a pelvic fracture model in a volume-dependent manner. Moreover, IH-PPB had no significant influence on bladder pressure in all four groups. Histological examination after the experiment presented no signs of injuries in the colon, rectum, or bladder.

Massive hemorrhage after a severe pelvic fracture necessitates emergency treatment to manage bleeding. Preperitoneal packing and arterial embolism have been established as efficient approaches to managing bleeding after severe pelvic fracture (Cheng et al., 2018). Notwithstanding, the latter requires not only specialized equipment and a skilled interventionist but also a slightly extended duration of operation. The former appears to be the optimal emergency approach to controlling bleeding from severe pelvic fractures while awaiting transfer to definitive surgical hemostasis (Cheng et al., 2018).

Dr. Do has provided a potential alternative to the traditional preperitoneal packing, namely preperitoneal ballooning, to timely manage vascular bleeding in a swine model. The preperitoneal balloon was prepositioned in the preperitoneal space prior to inducing vascular injury in Dr. Do’s report. After injury to the external iliac artery and vein, the hematoma rapidly developed in the preperitoneal space. In fact, the preperitoneal balloon was located in the hematoma when the balloon was inflated after vascular injuries (Mirilas et al., 2005; Read, 2005; Ohuchi et al., 2019).

Moreover, hematoma is typically encountered during the placement of surgical packs during preperitoneal packing in the emergency department. The presence of a hematoma in the preperitoneal space could assist in controlling bleeding by compressing the injured vessel. In this study, the preperitoneal pressure was increased from 0 to 9.00 ± 1.41 mmHg after vascular injury, indicating the accumulation of hematoma in the preperitoneal space, leading to elevated pressure. Therefore, the hematoma collaborates with IH-PPB to facilitate hemostasis.

The ideal pressure to control bleeding and concomitantly avoid visceral damage in the colon, rectum, and bladder remains unknown. This study compared the efficacy of 1,000-, 800-, and 500-mL preperitoneal balloons in controlling hemorrhages from vascular injuries. Our results unveiled that the preperitoneal balloon controlled vascular bleeding after severe pelvic fracture in a volume-dependent manner. Notably, the 1000-mL and 800-mL preperitoneal balloons had similar efficacy in controlling vascular bleeding. What’s more, the bladder pressure, which reflects intra-peritoneal pressure, was comparable between the control and IH-PPB groups. Histological examination following HE staining also revealed no evidence of damage in the colon, rectum, and bladder. It suggested that IH-PPB volumes within 1,000 mL do not result in significant organ damage within the pelvic space.

This study found that higher-pressure compression promoted coagulation even in the presence of 5-mm tears adjacent to the external iliac arteries and veins. After 1 hour of observation, the preperitoneal balloons were deflated to assess hemostatic effects after preperitoneal balloon tamponade. Strikingly, the 1000-mL IH-PPB exhibited a 67% survival rate during the additional 10-min observation after balloon deflation. In 800-mL group, IH-PPB similarly displayed a 17% survival rate after balloon deflation. Laparotomy after euthanasia revealed that a blood clot had already formed and occluded the bleeding site after preperitoneal balloon tamponade. These observations suggested that preperitoneal balloons not only directly compress the vessels but also trigger intravascular coagulation to control bleeding.

The intra-hematoma preperitoneal balloon functions on a principle similar to that of a space occupation device, despite differences in shapes from that of the surgical packs. In traditional preperitoneal packing, the hematoma was recommended to be removed before placing the laparotomy pad. However, eliminating the hematoma not only wastes valuable time but also causes rebleeding after the removal of blood clots. Herein, the Bakri Balloon was placed before hematoma formation; hence, it was not a real-life situation. Therefore, we postulate that it is not necessary to remove the hematoma in an emergency setting. Importantly, the hematoma could indicate the source of bleeding. In the emergency department, the hematoma in the preperitoneal space can act as a marker, signifying the need for balloon insertion. A convenient balloon could be inserted into the hematoma in the preperitoneal space to control fatal bleeding during severe pelvic injuries under ultrasonic guidance in the future. Resuscitative endovascular balloon occlusion of the aorta (REBOA) is a damage control surgery for managing life-threatening hemorrhage in cases of severe pelvic fractures (Kam et al., 2019). Consequently, REBOA in zone 3 could be an effective alternative to preperitoneal balloon tamponade (PPP) in severe pelvic fractures (Do et al., 2019b). At present, REBOA combined with preperitoneal tamponade was recommended to treat severe pelvic fractures with greater hemodynamic instability in the clinic (Werner et al., 2022).

There were three major limitations in this study that cannot be overlooked. To begin, this study is a non-realistic situation wherein the time spent for placement of a preperitoneal balloon was not considered. In other words, the preperitoneal balloon was prepositioned in the preperitoneal space prior to vascular injury. However, the preperitoneal balloon cannot be immediately placed after vascular injury. Secondly, swine possess a more robust coagulation system than humans, given that their platelet count is almost double that of humans, and their activated partial thromboplastin time (APTT) is half that of humans (Stettler et al., 2017; Lechner et al., 2019). Hence, the data in this study may not accurately reflect the response to vascular injury in humans. Thus, caution is warranted when extrapolating these findings to human pelvic fractures. Thirdly, lower limb perfusion/ischemia was not monitored in this study. Although the experiment lasted only 60 min, which is generally considered safe for limb surgeries in the clinical setting, it is still necessary to evaluate the possibility of ischemia in the hind limbs. Lastly, the animal model could not simulate a real severe pelvic fracture because separation of pubic symphysis does not significantly expand its volume since swine pelvis is too smaller than human being and external iliac vessels, not internal iliac vessels which were usually damaged in pelvic fracture, were subjected to injury in our experiments in this study.

In summary, this study revealed that 800-mL and 1000-mL IH-PPB not only efficiently improved survival time and rate during the 60-min observation but also limited blood loss over the 60-min compression period. Moreover, 1000-mL IH-PPB was superior to 800-mL IH-PPB in terms of hemodynamic stability for the management of vascular bleeding. Altogether, IH-PPB may be a promising method to control vascular bleeding in severe pelvic fractures in pre-hospital or in-hospital emergencies while awaiting transfer to definitive surgical hemostasis.

## Data Availability

The original contributions presented in the study are included in the article/Supplementary material, further inquiries can be directed to the corresponding authors.
